# Comparison of inflammatory and ECG markers between children/adolescents with ADHD and healthy controls

**DOI:** 10.3389/fpsyt.2026.1704757

**Published:** 2026-03-31

**Authors:** Bari Ay, Umut Balatacı

**Affiliations:** Department of Child and Adolescent Psychiatry, Karabük University Faculty of Medicine, Karabük, Türkiye

**Keywords:** attention-deficit/hyperactivity disorder, frontal QRS-T angle, pan-immune-inflammation value (PIV), systemic immune-inflammation index (SII), systemic inflammation

## Abstract

**Background:**

Attention-deficit/hyperactivity disorder (ADHD) has been associated with low-grade systemic inflammation and autonomic dysregulation, but it remains unclear whether these alterations are accompanied by subclinical ventricular electrical heterogeneity in medicated pediatric patients. This study investigated inflammatory markers and the frontal QRS-T angle (fQRS-T) in children and adolescents with ADHD receiving methylphenidate and examined the association between inflammatory burden and fQRS-T.

**Methods:**

This single-center retrospective cross-sectional study included 75 children and adolescents with DSM-5 ADHD who had received continuous methylphenidate treatment for at least 6 months and 75 age- and gender-matched healthy controls. Participants with chronic inflammatory, autoimmune, cardiovascular, systemic, or psychiatric comorbidities were excluded. Complete blood count-derived inflammatory indices, including the systemic immune-inflammation index (SII), systemic inflammation response index (SIRI), and pan-immune-inflammation value (PIV), were calculated. Standard 12-lead electrocardiograms were used to assess heart rate, QT, QTc, QRS duration, and fQRS-T.

**Results:**

The ADHD and control groups were similar in age and gender distribution. Conventional ECG parameters, including heart rate, QT, QTc, and QRS duration, did not differ significantly between groups. In contrast, the fQRS-T angle was significantly wider in the ADHD group than in controls (31.05° ± 32.03° vs. 18.62° ± 20.02°; *p* = 0.038). Among inflammatory measures, neutrophil count, SII, and PIV were significantly higher in the ADHD group. Within the ADHD group, fQRS-T was positively correlated with SII (*r* = 0.363, *p* = 0.030) and treatment duration (*r* = 0.340, *p* = 0.036). Treatment duration was also positively correlated with SII (*r* = 0.322, *p* = 0.040). In linear regression analysis, both SII (*B* = 0.012, 95% CI: 0.002 to 0.022; *p* = 0.019) and treatment duration (*B* = 0.945, 95% CI: 0.203 to 1.687; *p* = 0.014) were associated with fQRS-T.

**Conclusion:**

Children and adolescents with ADHD receiving methylphenidate showed a higher inflammatory burden and a wider fQRS-T angle than healthy controls. The association of fQRS-T with SII suggests a possible link between low-grade systemic inflammation and subclinical ventricular electrical heterogeneity in this population. However, because of the retrospective cross-sectional design and the inclusion of only methylphenidate-treated patients, these findings should be interpreted cautiously and considered hypothesis-generating.

## 1 Introduction

Attention-deficit/hyperactivity disorder (ADHD) is a common neurodevelopmental condition marked by inattention, hyperactivity, and impulsivity, affecting roughly 5% of school-age children, with diagnoses more frequent in boys and typically made in the early school years (1). ADHD often co-occurs with disruptive, anxiety, or learning problems and may persist into adolescence, generating a sustained need for monitoring and treatment (2). Pharmacotherapy—mainly psychostimulants in our setting, alongside atomoxetine—is widely used and generally effective, but it also raises interest in cardiovascular and metabolic safety, prompting professional societies (e.g., AAP, AACAP) to recommend baseline and periodic electrocardiogram (ECG) and laboratory assessments in selected patients, especially when there is a cardiac family history (3, 4).

Beyond its behavioral and cognitive manifestations, ADHD has increasingly been discussed as a condition that may be accompanied by low-grade systemic inflammation, with reports of altered neutrophil–lymphocyte profiles and immune–metabolic signals in affected youth (5, 6). However, most available studies have focused on single hematologic ratios (5–7), have not evaluated composite CBC-derived indices such as the systemic immune-inflammation index (SII), systemic inflammation response index (SIRI), or pan-immune-inflammation value (PIV), and have rarely examined these together with electrocardiographic markers.

Frontal QRS-T angle (fQRS-T) is a simple, vector-based ECG parameter that reflects ventricular electrical heterogeneity and has prognostic value in cardiovascular disorders in several populations (8), yet it has been scarcely studied in pediatric neurodevelopmental disorders. To our knowledge, no study has jointly assessed CBC-derived inflammatory indices and fQRS-T in children/adolescents with ADHD receiving stimulant treatment. We therefore conducted a single-center case–control study comparing medicated ADHD patients with age- and gender-matched healthy controls to: (i) determine whether ADHD is associated with higher SII/SIRI/PIV and a wider fQRS-T; and (ii) explore correlations between inflammatory indices and fQRS-T. We hypothesized that medicated youth with ADHD would show a more proinflammatory peripheral profile and greater ventricular electrical heterogeneity than controls, and that inflammation would correlate positively with fQRS-T.

## 2 Methods

### 2.1 Participants and procedure

This study was designed as a retrospective, cross-sectional investigation. It was conducted at the Department of Child and Adolescent Psychiatry of Karabük Training and Research Hospital, Türkiye. Ethics approval was obtained from the Karabük University Non-Interventional Clinical Research Ethics Committee (Date: 28 July 2025; Decision No: 2025/2428). All procedures were carried out in accordance with the Declaration of Helsinki. Parents of all participants provided written informed consent prior to data extraction from medical records.

All children and adolescents who presented to the Child and Adolescent Psychiatry Outpatient Clinic between 1 August 2024 and 1 August 2025 and received a DSM-5-based diagnosis of ADHD were screened. From this pool, the ADHD group was formed by including those who were of an age consistent with the study period and had complete medical records; had a clearly documented ADHD diagnosis established according to DSM-5 criteria by a child and adolescent psychiatrist and supported by the Schedule for Affective Disorders and Schizophrenia for School-Age Children-Present and Lifetime Version (K-SADS-PL); had been on continuous methylphenidate treatment for at least 6 months before the ECG and laboratory assessment; had a mean daily methylphenidate dose of 40 mg ± 14 mg, with 25 participants receiving immediate-release formulations and 50 receiving extended-release formulations; had, during the same visit, routine laboratory tests (complete blood count and biochemistry) and a standard 12-lead ECG available in the hospital information system; had no chronic inflammatory, autoimmune, cardiovascular, or other systemic disease; had no comorbid psychiatric disorder; were not on any regular psychoactive or anti-inflammatory medication other than methylphenidate; and had a normal physical examination and resting ECG at the time of recording. Among the ADHD group, 35 participants (46.7%) had the combined presentation, 25 (33.3%) had the predominantly inattentive presentation, and 15 (20.0%) had the predominantly hyperactive/impulsive presentation.

Patients were excluded if they (i) had any medical condition that could affect cardiac function or hematologic/biochemical measurements (e.g., congenital heart disease, arrhythmia, anemia requiring treatment), (ii) had missing ECG or laboratory data, or (iii) declined use of their data.

**Figure 1:**
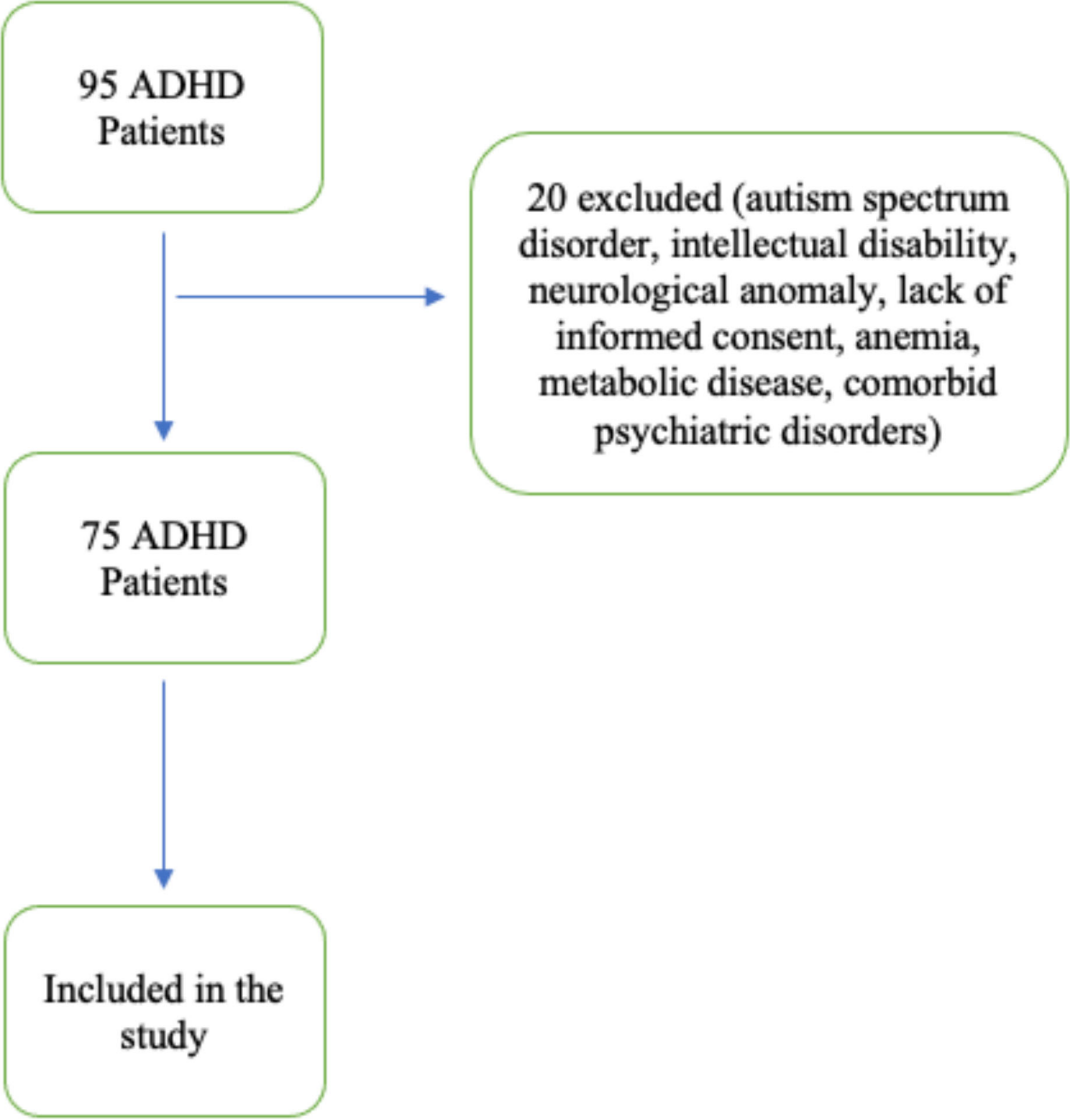
Flowchart of the study.

Healthy controls were recruited from the same hospital catchment area during the 1-year study period. They were frequency-matched to the ADHD group by age (± 6 months) and gender. Eligibility for controls required: (i) no psychiatric diagnosis on psychiatric evaluation supported by K-SADS-PL, (ii) no chronic inflammatory, autoimmune, or cardiovascular disease, (iii) no regular use of psychoactive or anti-inflammatory medication, and (iv) normal physical examination and resting ECG. Chart review confirmed that none of the included controls had psychiatric or medical comorbidities. The flowchart of the study group is shown in **Figure 1**.

### 2.2 Measures

A sociodemographic data form was developed for this study; it assessed age, gender, family structure, educational status, and variables related to psychiatric consultation.

#### 2.2.1 Blood analyses

Complete blood counts were performed on a Mindray BC-600 analyzer (Shenzhen Mindray Bio-Medical Electronics Co. Ltd., Shenzhen, China). Serum biochemistry was measured on an Atellica system (Siemens Healthineers, Erlangen, Germany). Hormone assays were run on an ADVIA Centaur XPT platform (Siemens Healthineers, Erlangen, Germany). The following indices were calculated: systemic immune-inflammation index (SII) = platelet × neutrophil ÷ lymphocyte; systemic inflammation response index (SIRI) = neutrophil × monocyte ÷ lymphocyte; and pan-immune-inflammation value (PIV) = platelet × neutrophil × monocyte ÷ lymphocyte.

#### 2.2.2 ECG acquisition and processing

Standard 12-lead ECGs were recorded using the same device (MAC 2000, GE Healthcare, Chicago, IL, USA) in the supine position, at a paper speed of 25 mm/s and a calibration of 10 mm/mV, with standard lead placement. Tracings with artifacts, lead misplacement, or poor quality were excluded.

**Figure 2:**
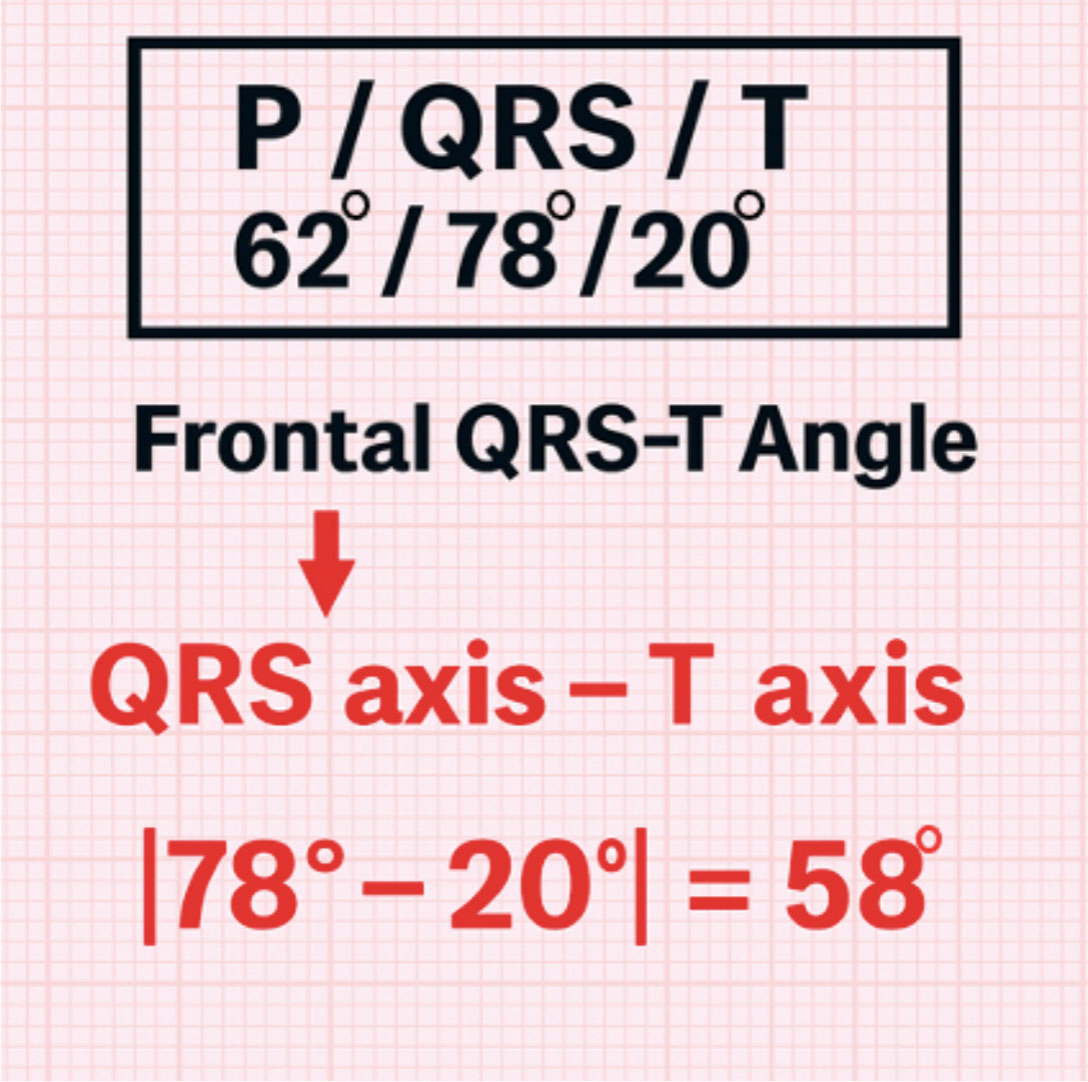
Calculation of the fQRS-T.

The QT interval was measured in milliseconds from the onset of the QRS complex to the end of the T wave. The end of the T wave was determined using the tangent method (tangent to the terminal downslope intersecting the isoelectric line). If lead II was unsuitable, lead V5 or another lead with the clearest T wave was used. Measurements were averaged over three consecutive sinus beats. QRS duration was measured from the earliest QRS onset in any lead to the latest J-point/return to baseline across the 12 leads; the maximum value across leads was taken and averaged over three beats. Heart-rate-corrected QT (QTc) was calculated using Bazett’s formula (QTc = QT/√RR), with RR taken as the mean of three sinus cycles, and is reported in milliseconds (9). The fQRS-T angle was obtained from the automated 12-lead ECG report as the absolute difference between the frontal QRS axis and the T-wave axis (range 0°–180°). When the absolute difference exceeded 180°, it was converted using the conventional formula: 360° − |QRS axis − *T* axis| (10). This calculation is illustrated in **Figure 2**.

All ECG tracings and automated measurements were independently reviewed by a cardiologist who was blinded to group allocation (ADHD vs. control), clinical characteristics, and laboratory results. In cases of discrepancy between automated and visual measurements, the cardiologist’s measurement was accepted.

### 2.3 Data analysis

Descriptive statistics were calculated for all variables. Categorical variables were presented as frequency (*n*) and percentage (%). Normality was assessed with the Kolmogorov–Smirnov test. Between-group comparisons of categorical variables were performed using Pearson’s Chi-square or Fisher’s exact test, as appropriate. Variables that approximated a normal distribution were presented as mean ± SD, whereas nonnormally distributed variables were presented as median (IQR, 25th–75th percentile). For two-group comparisons, Student’s *t*-test was used for normally distributed variables and the Mann–Whitney *U* test for nonnormally distributed variables. Spearman’s *r* was used to examine correlations between variables. Cohen’s *d* was reported for variables summarized as mean ± SD. A *p*-value < 0.05 was considered statistically significant. Analyses were conducted using IBM SPSS Statistics for Mac, Version 26.0 (IBM Corp., Armonk, NY, USA).

## 3 Results

The ADHD (*n* = 75) and healthy control (HC, *n* = 75) groups were comparable in age (10.97 years ± 2.59 years vs. 11.74 years ± 3.32 years; *p* = 0.247) and gender distribution (male subjects: 77.3% vs. 69.3%; *p* = 0.383). No significant baseline differences were observed (**Table 1**).

**Table 1:** Baseline demographic characteristics of the study groups.

Variable	ADHD (*n* = 75)	HC (*n* = 75)	*p*-value
Age (years)	10.97 ± 2.59	11.74 ± 3.32	0.247
Sex (*n*, %)			
Male	58 (%77.3)	52 (%69.3)	0.383
Female	17 (%22.7)	23 (%30.7)	

Data are mean ± SD or *n* (%).

*ADHD*, attention-deficit/hyperactivity disorder; *HC*, healthy controls.

Comparison of ECG parameters between the groups is shown in **Table 2**. The fQRS-T angle was significantly larger in the ADHD group (31.05° ± 32.03°) than in the HC group (18.62° ± 20.02°), with a mean difference of 12.43° (95% CI, 0.69 to 24.17; *p* = 0.038) and a moderate effect size (Cohen’s *d* = 0.47). Despite considerable within-group variability, this finding suggests greater ventricular depolarization–repolarization mismatch in the ADHD cohort, whereas conventional intervals (QT, QTc, and QRS duration) were comparable between groups. The clinical relevance of the wider fQRS-T angle warrants confirmation in larger samples with attention to potential modifiers such as age distribution and medication status.

**Table 2:** Electrocardiographic parameters in ADHD vs. healthy controls.

Variable	ADHD (*n* = 75)	HC (*n* = 75)	Mean difference (95% CI)	Cohen’s *d*	*p*-value
Heart rate (bpm)	85.41 ± 16.99	84.19 ± 16.94	1.22 (− 4.25 to 6.69)	0.07	0.750
QT (ms)	357.78 ± 29.15	355.30 ± 31.72	2.48 (− 7.14 to 12.10)	0.07	0.718
QTc (ms)	421.37 ± 24.03	415.05 ± 20.95	6.32 (− 3.47 to 16.11)	0.28	0.212
QRS duration (ms)	80.13 ± 8.64	80.02 ± 9.40	0.11 (− 2.79 to 3.01)	0.01	0.956
fQRS-T (deg)	31.05 ± 32.03	18.62 ± 20.02	12.43 (0.69 to 24.17)	0.47	**0.038**

Values are presented as mean ± SD. Mean differences are shown with two-sided 95% confidence intervals (CIs). Cohen’s *d* was used to estimate effect size, and *p*-values were obtained from independent-samples tests. Units are bpm for heart rate, ms for intervals, and degrees for fQRS-T. *ADHD*, attention-deficit/hyperactivity disorder; *HC*, healthy controls; *QTc*, corrected QT interval; *fQRS-T*, frontal QRS-T angle.

The bold values indicate statistically significant results, defined as p < 0.05.

Comparison of laboratory and inflammatory parameters between the groups is shown in **Table 3**. Neutrophil counts were higher in the ADHD group, with a median-based difference of 0.55 (95% CI, 0.05 to 1.05; *p* = 0.031). Among the composite inflammatory indices, SII and PIV were also significantly higher in ADHD, with effect estimates of 128.50 (95% CI, 40.20 to 216.80; *p* = 0.005) and 64.10 (95% CI, 6.70 to 121.50; *p* = 0.030), respectively.

**Table 3:** Laboratory parameters in ADHD vs. healthy controls.

Variable	ADHD (*n* = 75)	HC (*n* = 75)	Effect estimate (95% CI)	Cohen’s *d*	*p*-value
Hgb (g/dL)	13.20 (12.12–14.15)	12.90 (12.50–13.95)	0.10 (− 0.20 to 0.40)	–	0.602
WBC (10^9^/L)	7.50 (5.30–8.70)	6.55 (5.40–8.30)	0.35 (− 0.45 to 1.10)	–	0.437
Platelets (10^9^/L)	302.28 ± 60.78	277.19 ± 73.34	25.09 (− 5.70 to 55.88)	0.37	0.108
Neutrophils (10^9^/L)	4.35 (3.25–5.25)	3.55 (2.90–4.28)	0.55 (0.05 to 1.05)	–	**0.031**
Lymphocytes (10^9^/L)	2.04 ± 0.51	2.28 ± 0.83	− 0.24 (− 0.56 to 0.08)	− 0.35	0.135
Monocytes (10^9^/L)	0.45 (0.38–0.54)	0.41 (0.37–0.55)	0.02 (− 0.04 to 0.07)	–	0.605
Basophils (10^9^/L)	0.1 (0.1–0.1)	0.1 (0–0.1)	0.00 (− 0.01 to 0.01)	–	0.832
Eosinophils (10^9^/L)	0.17 (0.09–0.24)	0.15 (0.11–0.26)	0.01 − 0.03 to 0.05)	–	0.752
Glucose (mg/dL)	89.73 ± 9.99	90.61 ± 9.27	− 0.88 (− 3.98 to 2.22)	− 0.09	0.713
Urea (mg/dL)	23.62 ± 6.98	24.86 ± 8.21	− 1.24 (− 3.70 to 1.22)	− 0.16	0.524
Creatinine (mg/dL)	0.57 ± 0.11	0.64 ± 0.18	− 0.07 (− 0.14 to 0.00)	− 0.47	0.063
AST (U/L)	25.08 ± 5.18	24.26 ± 8.37	0.82 (− 1.60 to 3.24)	0.12	0.657
ALT (U/L)	18.15 ± 5.74	17.87 ± 8.74	0.28 (− 2.05 to 2.61)	0.04	0.882
TSH (uIU/mL)	1.89 (1.23–2.44)	1.97 (1.55–2.78)	− 0.08 (− 0.32 to 0.14)	–	0.326
fT4 (ng/dL)	1.19 ± 0.20	1.19 ± 1.13	0.00 (− 0.26 to 0.26)	0.00	0.935
Albumin (g/dL)	4.79 ± 0.30	4.75 ± 0.30	0.04 (− 0.06 to 0.14)	0.13	0.655
SII	599.86 (392.74–924.38)	462.25 (311.86–583.15)	128.50 (40.20 to 216.80)	**–**	**0.005**
SIRI	0.88 (0.61–1.42)	0.70 (0.49–1.13)	0.11 (− 0.01 to 0.24)	–	0.067
PIV	282.59 (172.14–425.79)	212.04 (115.14–309.29)	64.10 (6.70 to 121.50)	–	**0.030**

Data are mean ± SD or median (IQR). For approximately normally distributed variables, effect estimates are mean differences with 95% confidence intervals (CIs), and effect sizes are expressed as Cohen’s *d*. For nonnormally distributed variables, effect estimates are median-based differences with 95% CIs. Statistical inference (significance testing) follows the *p*-values reported in the original analysis. *ADHD*, attention-deficit/hyperactivity disorder; *HC*, healthy controls; *Hgb*, hemoglobin; *WBC*, white blood cells; *AST*, aspartate aminotransferase; *ALT*, alanine aminotransferase; *TSH*, thyroid-stimulating hormone; *fT4*, free thyroxine; *SII*, systemic immune-inflammation index; *SIRI*, systemic inflammation response index; *PIV*, pan-immune-inflammation value; *SD*, standard deviation; *IQR*, interquartile range.

The bold values indicate statistically significant results, defined as p < 0.05.

The correlations between inflammatory parameters, fQRS-T angle, and treatment duration are presented in **Table 4**. The fQRS-T angle was significantly positively correlated with SII (*r* = 0.363, *p* = 0.030) and treatment duration (*r* = 0.340, *p* = 0.036). In addition, treatment duration was significantly positively correlated with SII (*r* = 0.322, *p* = 0.040). No significant correlations were found between fQRS-T angle or treatment duration and SIRI or PIV.

**Table 4:** Examining the correlation between inflammatory parameters, fQRS-T, and treatment duration.

Variable	fQRS-T		Treatment duration	
	*p*-value	*r*	*p*-value	*r*
SII	**0.030**	**0.363**	**0.040**	**0.322**
SIRI	0.117	0.266	0.128	0.238
PIV	0.074	0.302	0.095	0.280
Treatment duration	0.036	0.340	N/A	N/A

*fQRS-T*, frontal QRS-T angle; *SII*, systemic immune-inflammation index; *SIRI*, systemic inflammation response index; *PIV*, pan-immune-inflammation value.

The bold values indicate statistically significant results, defined as p < 0.05.

**Figure 3:**
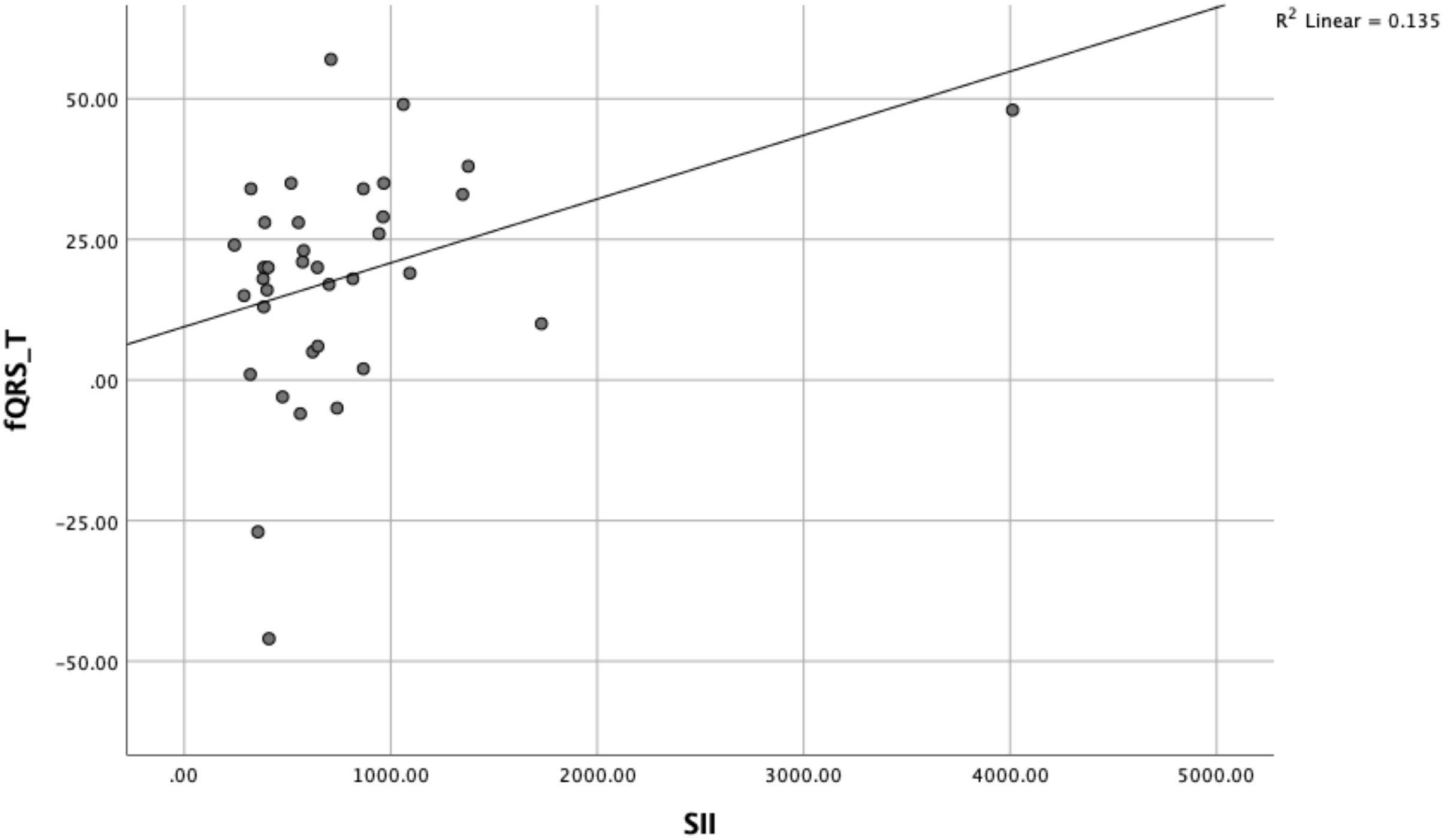
Correlation between SII and fQRS-T angle.

Scatter plot showing the positive correlation between SII and the fQRS-T angle (*r* = 0.363, *p* = 0.030; *R*^2^ = 0.135) (**Figure 3**).

Multiple linear regression analysis was performed with the fQRS-T angle as the dependent variable (see **Table 5**). SII, age, gender, and treatment duration were entered as independent variables. SIRI and PIV were excluded from the multivariable model because of multicollinearity. In the final model, SII (*B* = 0.012, 95% CI: 0.002 to 0.022, *β* = 0.318, *p* = 0.019) and treatment duration (*B* = 0.945, 95% CI: 0.203 to 1.687, *β* = 0.296, *p* = 0.014) remained significantly associated with the fQRS-T angle, whereas age and gender were not significant predictors.

**Table 5:** Multivariable linear regression analysis for factors associated with the fQRS-T angle.

Variable	*B*	Standard error	Standardized *β*	95% CI for *B*	*t*	*p*-value
SII	0.012	0.005	0.318	0.002 to 0.022	2.41	**0.019**
Age	0.847	0.624	0.156	− 0.401 to 2.095	1.36	0.179
Sex (male)	3.214	2.487	0.142	− 1.759 to 8.187	1.29	0.201
Treatment duration	0.945	0.371	0.296	0.203 to 1.687	2.55	**0.014**

Multivariable linear regression was used. *fQRS-T*, frontal QRS-T angle; *SII*, systemic immune-inflammation index. Model statistics: *R*^2^ = 0.286, adjusted *R*^2^ = 0.244, *p* = 0.006.

The bold values indicate statistically significant results, defined as p < 0.05.

## 4 Discussion

In this cross-sectional analysis of children and adolescents with ADHD who had been on methylphenidate for ≥ 6 months, we identified three related findings: (i) ADHD participants showed a more proinflammatory profile than age- and gender-matched healthy controls, mainly due to higher neutrophil counts and elevated SII and PIV; (ii) the ADHD group had a wider fQRS-T despite comparable conventional ECG intervals (QT, QTc, QRS); and (iii) SII was positively associated with fQRS-T, with SIRI and PIV showing similar but weaker trends. Taken together, these observations suggest that, among children and adolescents with ADHD in this medicated cohort, low-grade systemic inflammation may be associated with subtle electrical heterogeneity detectable on a standard 12-lead ECG.

Our inflammatory findings are consistent with a growing body of pediatric literature showing that CBC-derived indices such as neutrophil-to-lymphocyte ratio (NLR), platelet-to-lymphocyte ratio (PLR), and especially the composite systemic immune–inflammation index (SII) tend to be higher in ADHD than in healthy controls, and that PIV has recently emerged as a potentially sensitive marker in child and adolescent neuropsychiatric samples. Meta-analytic data reporting elevated NLR and PLR in ADHD accord with our observation that neutrophil-weighted composites (SII, PIV) were higher in the ADHD group (11). Similarly, a 2025 pediatric study highlighted SII as reflecting systemic inflammation in ADHD, noting that many participants were methylphenidate-treated, which parallels our cohort (12). A 2023 pediatric investigation also reported diagnostic value for SII and PIV in ADHD using ROC analyses, supporting the external face validity of our results (13). Because SII, SIRI, and PIV integrate granulocytic/monocytic and thrombocytic activity relative to lymphocyte counts, they may better reflect overall inflammatory tone than single-ratio metrics (14), and comparative work in other disease contexts suggests they can equal or outperform NLR/PLR while remaining inexpensive and CBC-based—an advantage in pediatrics (15).

From the electrophysiologic perspective, the fQRS-T angle quantifies the mismatch between ventricular depolarization and repolarization vectors and is regarded as a robust indicator of global electrical heterogeneity. It has been associated with adverse outcomes across multiple cardiac conditions—even when QTc is normal—particularly in adults, but also in older children. Our finding that fQRS-T was wider in the ADHD group, despite similar QT, QTc, and QRS intervals, fits this concept: vector discordance can be abnormal even when single ECG intervals appear normal. Adult studies have shown that both frontal and spatial QRS-T angles carry diagnostic and prognostic information for ventricular arrhythmias and mortality; pediatric reference data based on synthesized vectorcardiography have further defined age-related distributions, helping to contextualize what constitutes an “abnormal” angle in younger populations (16, 17). Although the present study was not designed for prognostication, the positive association between SII and fQRS-T suggests a potential relationship between systemic inflammation and electrical heterogeneity in ADHD.

Interpretation of the wider fQRS-T angle in our cohort also requires caution from a pediatric perspective. Although pediatric reference work has established age-related normal values for vectorcardiographic QRS-T angles, these data mainly concern the spatial QRS-T angle rather than the routinely reported frontal QRS-T angle used in the present study, and age-specific clinical cut-offs for fQRS-T in children and adolescents remain limited. Pediatric data suggest that QRS-T angle distributions vary with age, with changes occurring across childhood and adolescence. Therefore, while the higher mean fQRS-T value observed in our ADHD group may indicate greater electrical heterogeneity at the group level, it should not be interpreted as demonstrating a definite arrhythmic-risk threshold for an individual child. Moreover, although wider QRS-T angles are associated with adverse cardiovascular outcomes in adults and have shown potential prognostic value in selected pediatric cardiac populations, evidence directly linking mildly increased fQRS-T values to arrhythmia or cardiovascular events in otherwise healthy children and adolescents is still limited. Accordingly, our finding should be interpreted primarily as a marker of subclinical electrical heterogeneity rather than a proven indicator of immediate clinical risk (17–19).

The potential biological relevance of CBC-derived composite inflammatory indices in psychiatry is also supported by evidence beyond ADHD. For example, a longitudinal study in bipolar disorder reported that indices such as SII and SIRI varied in relation to illness phase and remission status, suggesting that these markers may reflect biologically meaningful inflammatory processes rather than nonspecific hematologic noise alone. Such findings strengthen the rationale for considering composite CBC-derived indices as accessible peripheral correlates of neuroimmune dysregulation across psychiatric disorders (20).

Several non-mutually exclusive mechanisms could explain why ADHD is accompanied by low-grade inflammation and why this, in turn, might relate to repolarization heterogeneity. Autonomic imbalance and stress-related physiology in ADHD may prime immune activation: reduced heart-rate variability (HRV), frequently described in ADHD, has been linked in meta-analytic work to higher inflammatory tone via the “inflammatory reflex” pathway, suggesting a bidirectional autonomic–immune loop (21). Neuroimmune studies have identified ADHD subgroups with elevated cytokines (e.g., IL-6, TNF-α), particularly when obesity or specific symptom dimensions are present; recent translational approaches have even proposed inflammatory “biotypes” of ADHD connected to chronic stress exposures, although results remain heterogeneous across cohorts and assays (22, 23). In parallel, behavioral/contextual factors common in ADHD—sleep disruption, irregular diet, sedentary behavior, and psychosocial stress—can amplify low-grade inflammation and shift leukocyte patterns toward neutrophil predominance, which SII and PIV capture by design (13). Within this framework, our finding of higher neutrophil counts and elevated SII/PIV in medicated ADHD youth is biologically plausible. SII emphasizes innate immune activation (neutrophils, platelets) relative to adaptive responses (lymphocytes); PIV additionally incorporates monocytes, which can increase in chronic low-grade inflammatory and endothelial states. Emerging pediatric psychiatric data likewise report SII/PIV elevations across several disorders (e.g., OCD, ADHD), supporting the idea that these multicell composites are sensitive markers of brain–body inflammatory coupling (24).

Several biologically plausible mechanisms may underlie the observed association between higher inflammatory indices and a wider QRS-T angle. Proinflammatory cytokines such as IL-6 and TNF-α can modulate ion-channel expression and function, calcium handling, and gap-junction integrity, thereby fostering spatial dispersion of repolarization. Low-grade inflammation can also promote microvascular dysfunction and interstitial alterations, subtly changing conduction velocity and action-potential–duration gradients and thus increasing vector discordance without necessarily prolonging the QT interval. Autonomic shifts—in particular sympathetic predominance and vagal withdrawal—may further increase repolarization heterogeneity through catecholaminergic effects on IKs and ICaL, which in aggregate widen the QRS-T angle. These mechanisms are compatible with adult and pediatric cardiology studies showing that wider QRS-T angles discriminate diseased from healthy states and correlate with arrhythmic burden in specific cardiomyopathies (16, 17, 25).

An important contextual factor in interpreting these findings is that all ADHD participants had been receiving methylphenidate for ≥ 6 months. Stimulants are known to produce modest, group-level increases in heart rate and blood pressure, but consistent QTc prolongation has not been demonstrated in pediatric meta-analyses or controlled trials—an observation that aligns with our finding of similar QT, QTc, and QRS values between groups and a difference only in fQRS-T (26–30). Nonetheless, methylphenidate has sympathomimetic and autonomic effects, and catecholaminergic pathways can influence both innate/adaptive immune activity, leukocyte trafficking, and ventricular repolarization heterogeneity; therefore, stimulant exposure could have contributed to the inflammatory pattern (elevated SII/PIV) and/or to the wider fQRS-T, independent of ADHD pathophysiology itself (12, 27, 31–33). In our additional analyses, treatment duration was positively correlated with both SII and fQRS-T angle, which further supports the possibility that treatment-related factors may have contributed to the observed associations. Because our design was cross-sectional and did not include a stimulant-naïve ADHD arm, we cannot disentangle disorder-related biology from treatment-related changes. Large, contemporary registry data likewise indicate that cumulative ADHD-medication exposure is associated with higher long-term cardiovascular risk—most evidently for methylphenidate and lisdexamfetamine—mainly through hypertension and arterial disease and with a duration–response pattern (34). Therefore, the cardio-immune pattern observed in the present study should be interpreted cautiously as hypothesis-generating and should be confirmed in prospective, stimulant-naïve cohorts with standardized covariates.

### 4.1 Limitations

This was a single-center, retrospective study, which limits causal inference and generalizability. A major limitation is that all ADHD participants had received methylphenidate for at least 6 months. Consequently, treatment effects cannot be separated from ADHD-related biology, and both the inflammatory findings and the wider fQRS-T angle may partly reflect stimulant exposure rather than the underlying disorder itself. Although treatment-related characteristics such as mean daily dose, formulation, and treatment duration were added in the revised manuscript, the absence of a stimulant-naïve ADHD group remains an important limitation. Moreover, several clinically relevant variables that may affect systemic inflammation—such as BMI/obesity, sleep disturbances, physical activity, dietary habits, exposure to cigarette smoke, and pubertal status—were not systematically available in the retrospective records and could not be adjusted for in the analyses. Accordingly, residual confounding cannot be excluded. These findings should therefore be considered hypothesis-generating and confirmed in larger, prospective studies including medication-naïve patients and standardized assessment of inflammatory confounders.

## 5 Conclusion

Medicated children and adolescents with ADHD showed higher SII/PIV values and a wider fQRS-T angle than healthy controls, and SII was positively associated with fQRS-T. Taken together, these findings suggest a potential association between low-grade inflammation and subclinical ventricular electrical heterogeneity in ADHD. The cross-sectional design and universal stimulant exposure preclude causal interpretation. Replication in larger, prospective, stimulant-naïve samples is needed before any clinical application can be considered.

## Data Availability

The raw data supporting the conclusions of this article will be made available by the authors, without undue reservation.
